# Social Media–Promoted Weight Loss Among an Occupational Population: Cohort Study Using a WeChat Mobile Phone App-Based Campaign

**DOI:** 10.2196/jmir.7861

**Published:** 2017-10-23

**Authors:** Chao He, Shiyan Wu, Yingying Zhao, Zheng Li, Yanyan Zhang, Jia Le, Lei Wang, Siyang Wan, Changqing Li, Yindong Li, Xinying Sun

**Affiliations:** ^1^ Department of Health Education, Shunyi Center for Disease Prevention and Control Beijing China; ^2^ Department of Social Medicine and Health Education, School of Public Health, Peking University Health Science Center Beijing China

**Keywords:** WeChat, weight loss, social media, health, intervention

## Abstract

**Background:**

Being overweight and obese are major risk factors for noncommunicable diseases such as cardiovascular diseases. The prevalence of overweight and obesity is high throughout the world and these issues are very serious in the Shunyi District in China. As mobile technologies have rapidly developed, mobile apps such as WeChat are well accepted and have the potential to improve health behaviors.

**Objective:**

This study aims to evaluate the effectiveness of a mobile app (WeChat) as an intervention on weight loss behavior.

**Methods:**

This study was conducted among an occupational population from August 2015 to February 2016 in the Shunyi District of Beijing. Before the intervention, the Shunyi District Government released an official document for weight loss to all 134 government agencies and enterprises in Shunyi District. Participants willing to use our official WeChat account were enrolled in a WeChat group and received 6 months of interventions for weight loss; those who were not willing to use the account were in a control group given routine publicity on weight loss.

**Results:**

In total, 15,310 occupational participants including 3467 participants (22.65%) in the control group and 11,843 participants (77.35%) in the WeChat group were enrolled. Participants in the WeChat group lost more weight (mean 2.09, SD 3.43 kg) than people in the control group (mean 1.78, SD 2.96 kg), and the difference in mean weight loss between the two groups for males was significant based on the stratification of age and educational level. To control for confounding factors and to explore the effects of WeChat on weight loss, the propensity score method with a multinominal logistic regression was utilized. For males, this showed that the WeChat group (with both active and inactive subgroups) had a higher probability of maintaining weight, weight loss from 1 to 2 kg, or weight loss more than 2 kg than the control group. However, the control group had higher probability of weight loss from 0 to 1 kg. Being active in WeChat was likely to be associated with weight loss. The more active participants were in the weight loss program via WeChat, the more weight they lost.

**Conclusions:**

The weight loss intervention campaign based on an official WeChat account focused on an occupation-based population in Shunyi District was effective for males. The more active male participants were in using WeChat, the more weight they lost. There might be no effect or there may even be a negative effect on weight loss for females. Future research should focus on how to improve adherence to the WeChat weight loss interventions, to improve and refine the WeChat content such as developing a variety of materials to attract interest, and to protect personal privacy, especially for females.

## Introduction

Being overweight is defined as having a body mass index (BMI) of 25 kg/m^2^ or higher; a person with a BMI of 30 kg/m^2^ or higher is regarded as obese. Worldwide in 2014, more than 1.9 billion adults were overweight and more than 600 million adults were obese. The percentage of obesity in the world’s adult population was 13%. The prevalence of obesity in 2014 was twice that of 1980 [1]. In China in 2013, 30.1% of adults were overweight and 11.9% of adults were obese, an increase of 7.3% and 4.8%, respectively, from 2002 [2]. Shunyi District is located in the northeast part of Beijing City. Its economy has developed rapidly because the Beijing Capital International Airport is located there. In Shunyi District in 2014, 38.3% of adults were overweight and 27.1% of adults were obese [3]. The issues of being overweight and being obese in Shunyi District are very serious.

Obesity is a major risk factor for noncommunicable diseases such as diabetes, cardiovascular diseases, and some cancers [4,5]. Obesity is a multifactorial and complex condition. Environmental factors, endocrine and inflammatory pathways, and endogenous genetic factors have effects on the development of obesity and obesity-related diseases [4]. Additionally, some demographic characteristics, such as age, education level, and social support, are associated with obesity. Older people, with lower education levels, lack of social support, and psychosocial pressures are more likely to be obese. Compared with men, socioeconomic parameters are more strongly associated with obesity in women [6]. Some scholars even argue that obese people suffer from social discrimination in medical care, employment, and education settings [7]. Therefore, how to reduce these determinants has become an important question that has confused public health practitioners because people who are overweight or obese have great difficulty losing weight. Traditionally, calorie restriction, exercising more, and eating less fat are the most common methods for weight loss [8]. Although there were some traditional weight loss interventions, the prevalence of obesity remains high and is increasing. Additionally, a large number of people who are obese do not adhere to weight loss interventions. Thus, new interventions should be developed to help people lose weight.

Various media such as radio, television [9], and Web-based interventions [10] have been utilized for mass outreach health campaigns [11]. Social media can influence health knowledge, beliefs, and attitudes, and also health behaviors, especially for large groups of people [9]. Recently, with the dramatic growth of Web 2.0 technologies, online social networks have also grown and account for approximately 27.18% of all time spent online among Chinese college freshmen [12]. Because online social networks have several advantages, such as a large audiences, higher levels of user engagement [13], and higher retention levels of existing contacts [14], they might have numerous potential effects on changing health behaviors [11].

Social media interventions have measurable impacts on health outcomes compared with non-social media–based interventions [15]. For women, the more time spent on Facebook leads to more comparisons of body and weight, more attention to the physical appearance of others, and more negative body attitudes [16]. An average weight loss of 42.3 pounds has been reported since weight loss bloggers started to blog about their weight loss attempts, and weight loss during blogging can be predicted by blogging duration [17]. A mobile phone app known as With U that allows friends to challenge one another to lose weight by using offline social networks of friends and the online network Facebook was effective regarding both the motivation to lose weight and on the amount of weight lost [18]. For some participants, especially for regular users of social media, a private Twitter weight loss group was found to be feasible and acceptable in losing weight [19]. Compared with offline friends, family members, and Facebook friends, participants who use Twitter to discuss their weight loss are exposed to more sources of positive social influence and fewer sources of negative social influence regarding weight loss [20]. Women of childbearing age actively use Twitter and show great interest in Twitter-based weight loss interventions [21].

WeChat (the Chinese version is *Weixin*), the popular instant-messaging app created by China’s largest Internet company, Tencent, has been regarded as the best social networking site in China [22] and is used in more than 200 countries [23]. Throughout the world, its registered users and active users total more than 1.12 billion and 600 million, respectively [24]. WeChat, similar to Facebook, Twitter, and YouTube, is a platform where people of all ages and professions can work with others, find and share information, and so on [25]. As a representative form of modern messaging software, WeChat has been used to change health behaviors and has shown potential impacts. A health education program using an official WeChat account to improve malaria health literacy among Chinese expatriates was proven to be effective, sustainable, feasible, and well accepted [23]. Using the WeChat app for follow-up was time-effective, cost-effective, and convenient [26]. WeChat interventions were effective in improving patient compliance and in reducing the treatment duration of orthodontic treatment [27]. Other social media platforms such as Facebook and Twitter have been used for weight loss; however, little is known about whether WeChat can be just as effective. Therefore, we hypothesize that interventions via WeChat will help people who are overweight or obese to lose weight.

## Methods

### Design and Setting

This study was conducted on an occupation-based population from August 2015 to February 2016 in the Shunyi District of Beijing to explore the effectiveness of the mobile app WeChat on weight loss behavior. Participants who were willing to use our official WeChat account were enrolled in a WeChat group and received 6 months of interventions for weight loss, and those who were not willing to use the account were in the control group. Before the interventions began, the Shunyi District Government released an official weight loss document to all 134 government agencies and enterprises in Shunyi District. At least 60% of the staff of government agencies were required to participate in this activity. The government agencies included the Shunyi Branch of the Beijing Municipal Public Security Bureau, the Shunyi Court, the Shunyi People’s Procuratorate, the Shunyi Branch of the Beijing Administration for Industry and Commerce in the Urban Management Law Enforcement Bureau, the Shunyi State Administration of the Taxation of China, the Beijing Shunyi Local Taxation Bureau, and the Food and Drug Administration of Beijing Shunyi District. At least 30% of the staff of other units were required to participate as well.

### Measures

Interventions were given to the WeChat group through the WeChat app and routine publicity, such as the slogan “take the stairs and lose weight,” was given to the control group. Participants were asked to report their demographic characteristics such as gender, age, educational level, and telephone number online when they registered with our official WeChat account. Additionally, on average, two weight managers per agency were trained to obtain participants’ data on height, weight, and waist circumference before and after the interventions were initiated for both groups. The demographic characteristics of participants in the control group were collected by weight managers.

### Sample Selection

Participants were enrolled if they (1) were from one of any of the 134 government agencies and enterprises in Shunyi District, (2) were 18 years of age or older, and (3) wanted to lose weight. Participants willing to lose weight via WeChat were enrolled as members of the WeChat group and those who were willing to lose weight but did not want to use WeChat were in the control group. Pregnant women and those whose health conditions were not suitable for weight loss were excluded from the study.

### Ethics Statement

All participants gave verbal consent to participate in the study and they were entitled to withdraw from the study whenever they wished and for whatever reason. The official weight loss document also addressed all the individuals who enrolled or who dropped out of our study voluntarily. People in the WeChat group would scan a QR code, below which was the phrase “enrolled or dropped out voluntarily” and for people in the control group, before they enrolled, weight managers in each agency told them everything about our study and asked them if they were willing to enroll. From the perspective of each participant’s private information, an agreement was made between the Shunyi Center for Disease Prevention and Control (SYCDC) and a technology company that provided technological support to protect all private information. Only two people from the technology company and two people from the SYCDC knew all the information. Private information such as a telephone number or identification number was deleted when the data were analyzed.

### Official WeChat Account Development and Components

**Figure 1:**
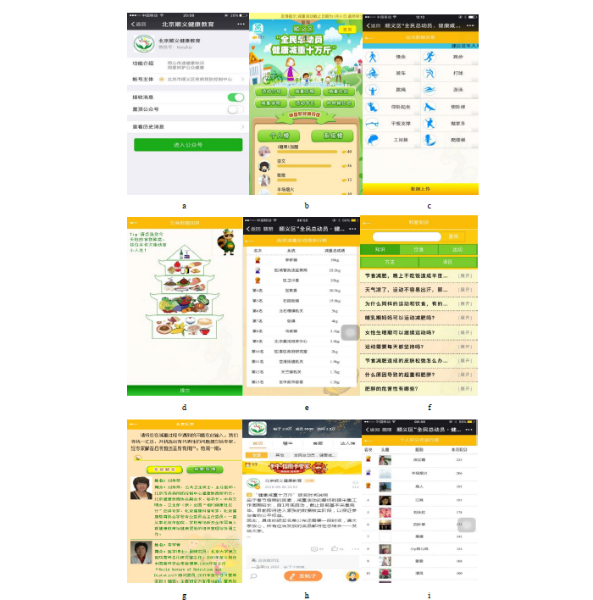
The interface of the WeChat interventions (originally in Chinese). (a) The official WeChat account “Health Education in Shunyi District, Beijing,” (b) introduction of the official account, (c) feedback on exercise, (d) feedback on diet, (e) the weight loss unit rankings, (f) the weight loss school, (g) the experts team, (h) the microcommunity, and (i) cumulative scores.

The SYCDC developed an official WeChat account, known as the “Health Education in Shunyi District, Beijing,” with the technological support of a specialized information technology company (Figure 1). Before becoming members of the official WeChat account, users were to register and provide information on their age, gender, educational level, and telephone number. Then, they were provided and could log in with a WeChat identification and password. All participants were asked to follow the instructions set forth in the official account after registration. Participants could read new messages and review the message history of all content published in the official account.

The official WeChat account consisted of the following six components: introduction, weight loss process, weight loss unit rankings, weight loss school, activity area, and awards (Figure 1). Each component is described in detail subsequently.

The weight loss process was designed to provide feedback on weight, diet, and exercise during the intervention process to motivate participants to lose weight. The duration of 12 types of activities, including walking, running, cycling, playing, skipping, swimming, sit-ups, push-ups, plank flat support, doing housework, workshops, and climbing stairs, were reported daily (Figure 1). According to a diet pagoda that was based on the Chinese Dietary Guidelines set forth by the Chinese Nutrition Society, each type of food was given a reasonable intake range; therefore, participants could know whether the amount of food they ate was excessive, moderate, or inadequate (Figure 1). Weight data were reported each week. When an individual reported data on diet, exercise, or weight, his or her physical condition would be assessed and the results would be provided immediately.

The weight loss unit rankings component aimed to rank the total score per unit, including the number of participants and the total amount of weight lost, to motivate and encourage participants to compete with one another (Figure 1). The weight loss school component consisted of a variety of materials on weight loss including micro videos and popular science knowledge (Figure 1) in addition to five experts (two from the Peking University Third Hospital, one from Peking University People’s Hospital, one from Peking University Health Science Center, and one from the Beijing Center for Disease Prevention and Control) who formed a consulting group to address questions from participants (Figure 1). The activity area component included a microcommunity (Figure 1) and other types of activities, where participants could communicate with one another. Finally, there was a rewards component. Participants received scores during the intervention and, to win a competition, participants had to lose weight and actively participate in various activities.

At the end of the program, winners and losers were determined on the basis of their cumulative scores (Figure 1). The scores could be from interactions, feedback information, reading articles, and so on. Different types of activities resulted in different scores. For example, if an individual registered with our official WeChat account, he or she would earn 10 points; reading articles earned two points per day and feedback information on weight, exercise, and diet were worth five points each. The top 60 WeChat active participants per month and the top 50 at the end of the project could win a prize.

### Content of WeChat Messages

A total of 210 messages were sent (one message every other day, on average) to participants in the intervention group during the period. The messages were read more than 247,000 times and were sent to other WeChat accounts more than 6500 times. In total, 3620 participants communicated with others in “microcommunity discussions” and there were more than 20,000 posts.

Participants in the intervention group could ask weight loss-related questions on our official WeChat account expert consultation page at any time during the 6-month period. Experts were consulted online more than 14,576 times and 28,000 questions were asked, among which 270 representative questions were responded to in detail. In addition, more than 8200 people regularly received feedback on their weight, diet, and exercise data and conducted self-assessments via WeChat. Examples of the content of WeChat messages can be found in Table 1.

**Table 1:** Examples of the content of WeChat messages.

Content examples^a^	Reading quantity	Number of forwards and favorites
Will beer increase your weight? Eight diet mistakes that will make you fatter!	2871	26
You do not have to go to the gym, seven other ways to burn fat	2945	44
Obesity is not an excuse to keep smoking	2982	24
Excessive weight will shorten your life! Eat less and exercise more to lose weight.	3060	60
Have you been cheated by the eight fallacies of weight loss?	3222	41
Want to have a good body image by spring? Do you know how to exercise now?	3233	24
New ways to lose weight. You do not need to diet.	3411	7
Five bad habits of running that will hurt your body!	3853	8
Do not miss the best season for weight loss! Weight loss is easier in winter!	4248	25
Changing these habits will keep you from regaining weight!	4577	28

^a^ Original text was in Chinese.

### Statistical Methods

Data was analyzed using SPSS version 18.0. Mean and standard deviation were used to present continuous variables for normal distribution or median and quartile for nonnormal distribution. Frequency and percentages were employed to express categorical variables. The chi-square test for categorical variables was used to compare parameters between the control group and the WeChat group, or *t* test for continuous variables with normal distribution. To control for confounding factors and to explore the effects of WeChat on weight loss, propensity score methods with a multinominal logistic regression was utilized; the propensity score replaced all single covariates to adjust the effectiveness on weight loss. A *P* value less than .05 was considered statistically significant.

## Results

### Participant Flow and Follow-Up

**Figure 2:**
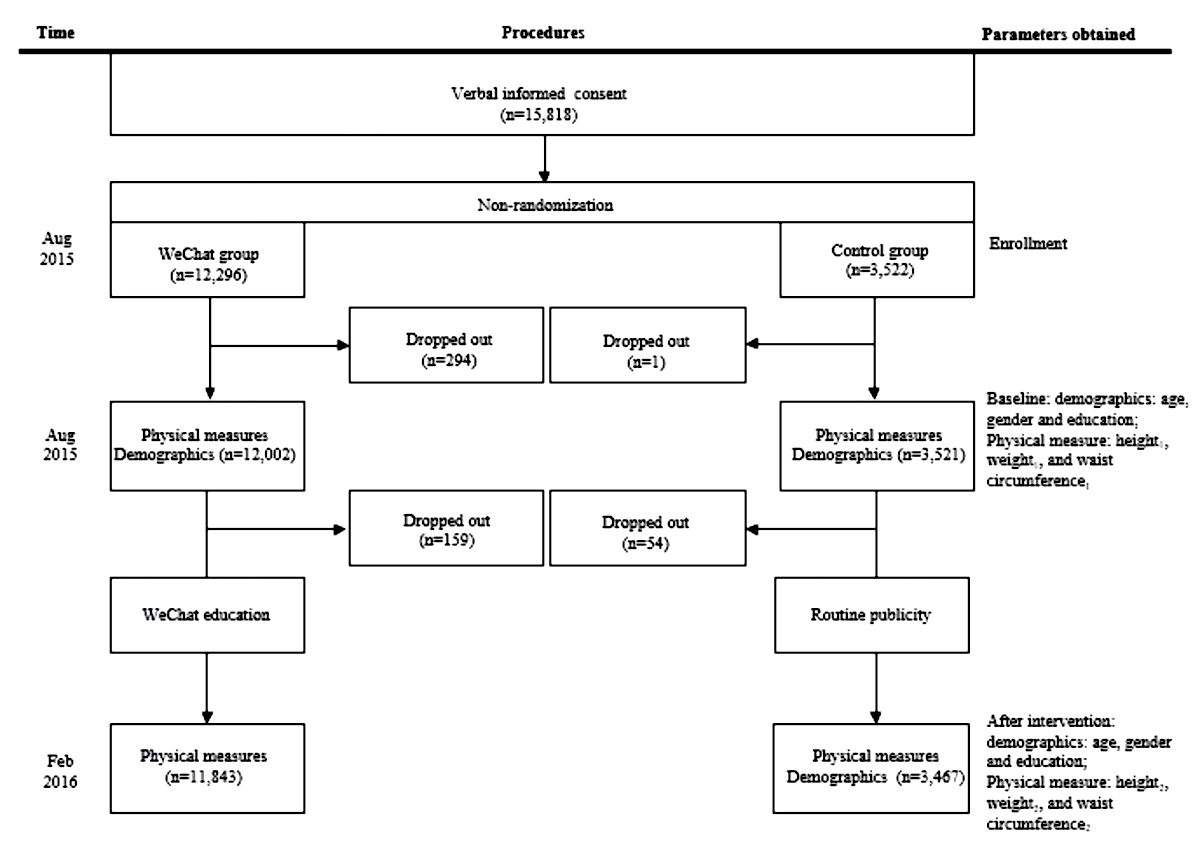
The flowchart of participation.

At the beginning, a total of 15,818 participants were enrolled, including 12,296 participants in the WeChat group and 3522 in the control group. Data were collected on a total of 15,523 participants, including 12,002 in the WeChat group and 3521 in the control group at the baseline. After 6 months of interventions, data were collected on a total of 15,310 participants, including 11,843 in the WeChat group and 3467 in the control group at the baseline and after 6 months of interventions (Figure 2).

### Participant Characteristics and WeChat Active

The data that were collected both at baseline and after 6 months of interventions were used. That is, a total of 15,310 participants were enrolled in this study. The mean age of the control group was 39.0 (SD 9.5) years and that of the WeChat group was 35.1 (SD 8.5) years. Participants in the WeChat group were younger than those in the control group, and the difference was significant. Participants in the WeChat group were largely females (66.53%, 7879/11,843) and largely had university/college degrees or above (91.85%, 10,878/11,843). In the control group, nearly half the participants were female (40.47%, 1403/3467) and more participants had university/college degrees or above. Baseline demographics including age, gender, and educational level between the two groups were not balanced (Table 2).

WeChat activeness was represented by WeChat cumulative scores, which were related to actual official WeChat account use. Higher scores indicated that an individual was more active in using WeChat to lose weight. Scores less than or equal to 50 were regarded as inactive, and scores of 50 or more were active. A significant number of participants in the WeChat group were inactive (83.18%, 9852/11,843) and only 16.8% (1991/11,843) were active. The differences between the control group and the WeChat group on demographics were all statistically significant (Table 2).

### Changes in Weight Loss Between the Control Group and the WeChat Group

The weight and waist circumference in the control group decreased by mean 1.78 (SD 2.96) kg and mean 2.39 (SD 3.91) cm, respectively, whereas in the WeChat group, weight and waist circumference decreased by mean 2.09 (SD 3.43) kg and mean 2.74 (SD 4.48) cm. A stratified analysis was performed to show the mean weight loss of the two groups. It also showed that for males, a decrease in weight loss was statistically significant, which indicated the 6-month WeChat interventions were effective for weight loss; however, for females, weight loss changes were not statistically significant (Table 3).

**Table 2:** Demographic characteristics of the control group and the WeChat group (N=15,310).

Demographics		Control group, n (%) (n=3,467)	WeChat group, n (%) (n=11,843)			χ^2^^a^	*P*^a^
			Inactive (n=9852)	Active (n=1991)	Total (n=11,843)		
**Gender**						798.0	<.001
	Male	2064 (59.53)	3415 (34.66)	549 (27.57) 3964 (33.47)			
	Female	1403 (40.47)	6437 (65.34)	1442 (72.43)	7879 (66.53)		
**Age group (years)**						443.1	<.001
	<40	1672 (50.61)	6,874 (70.32)	1180 (59.78)	8054 (68.55)		
	≥40	1632 (49.39)	2,901 (29.68)	794 (40.22)	3695 (31.45)		
**Education^b^**						536.9	<.001
	Low	772 (22.31)	779 (7.91)	186 (9.34)	965 (8.15)		
	High	2689 (77.69)	9073 (91.09)	1805 (90.66)	10,878 (91.85)		

^a^ Chi-square and *P* value are difference between the control group, the inactive group, and active group.

^b^ Low education level: high school or below; high education level: university/college or above.

**Table 3:** Mean weight loss in the two groups by gender, educational level, and age (N=11,530).

Gender, educational level,^a^ age, and groups				n	Mean (SD)	*t* (df)	*P*
**Male**							
	**Low educational level**						
		**<40 years**				3.36 (291)	.001
			Control	104	1.18 (4.51)		
			WeChat	189	3.22 (5.20)		
		≥ **40 years**				3.32 (485)	.001
			Control	274	1.95 (3.88)		^a^
			WeChat	213	3.27 (4.91)		
	**High educational level**						
		**<40 years**				8.10 (2231)	<.001
			Control	856	1.48 (2.79)		^a^
			WeChat	2405	2.51 (4.14)		
		≥ **40 years**				2.25 (1649)	.03
			Control 723	1.7 (2.82)			
			WeChat	1115	2.01 (3.12)		
**Female**							
	**Low educational level**						
		**<40 years**				1.95 (263)	.05
			Control	46	3.37 (5.26)		
			WeChat	219	2.08 (3.77)		
		≥ **40 years**				1.45 (540)	.15
			Control	237	2.57 (3.40)		
			WeChat	305 2.17 (2.96)			
	**High educational level**						
		**<40 years**				0.36 (914)	.72
			Control	666	1.98 (2.73)		
			WeChat	5241	1.94 (3.21)		
		≥ **40 years**				0.43 (622)	.66
			Control	392	1.87 (2.29)		
			WeChat	2062	1.82 (2.73)		

^a^ Low education level: high school or below; high education level: university/college or above.

### Estimating Adjusted WeChat Effectiveness

Propensity score methods are increasingly used to control for confounding factors in many medical studies. In our study, demographics including age, gender, and educational level between the two groups were not balanced at the baseline; thus, propensity score methods were used to control for them. With a group variable (the WeChat group and the control group) as the dependent variable and two demographic characteristics (age and educational level) as covariates, a binomial logistic regression analysis was used to estimate the propensity score based on gender.

To estimate WeChat effectiveness on weight loss, multinominal logistic regression used to test the parallel lines of the ordinal logistic regression that did not meet the criteria. The weight loss outcome was categorized as follows: weight gain, weight unchanged, weight loss from 0 to1 kilograms, weight loss from 1 to 2 kilograms, and weight loss more than 2 kilograms. The reference category for the dependent variable was the classification of weight gain. The propensity score was used as a continuous covariate replacing all single covariates with the independent variable, WeChat active group, in the multinominal logistic regression to estimate the adjusted WeChat effectiveness on weight loss.

For males, in the control group, 52.37% (1081/2064) of participants lost 0 to 1 kg, 35.72% (1220/3415) in the inactive group, and 41.17% (226/549) in the active group lost more than 2 kg. For females, 38.92% (546/1403) of participants in the control group and 31.30% (2015/6437) in the inactive group lost 0 to 1 kg and 33.36% (481/1442) in the active group lost more than 2 kg (Table 4).

**Table 4:** The frequency and percentage of weight loss outcomes between two groups based on gender.

Gender and weight		Control, n (%)	Inactivity, n (%)	Activity, n (%)	Total, n (%)
**Male**					
	Weight gain	187 (9.06)	272 (7.96)	45 (8.20)	504 (8.36)
	Weight unchanged	61 (2.96)	305 (8.93) 41 (7.47)	407 (6.75)	
	Weight loss (0-1 kg)	1081 (52.37)	932 (27.29)	136 (24.77)	2149 (35.65)
	Weight loss (1-2 kg)	219 (10.61)	686 (20.09)	101 (18.04)	1006 (16.69)
	Weight loss (≥2 kg)	516 (25.00)	1220 (35.72)	226 (41.17)	1962 (32.55)
**Female**					
	Weight gain	81 (5.77)	597 (9.27)	105 (7.28)	783 (8.44)
	Weight unchanged	77 (5.49)	732 (11.37)	112 (7.77)	921 (9.92)
	Weight loss (0-1 kg)	546 (38.92)	2015 (31.30)	427 (29.61)	2988 (32.19)
	Weight loss (1-2 kg)	259 (18.46)	1317 (20.46)	317 (22.00)	1893 (20.39)
	Weight loss (≥2 kg)	440 (31.36)	1776 (27.59)	481 (33.36)	2697 (29.06)

For males, the results of the multinominal logistic regression showed that when controlling for confounding factors, compared with the classification of weight gain, the WeChat group (with both active and inactive subgroups) had higher probability of maintaining weight, weight loss from 1 to 2 kg, or weight loss of more than 2 kg than the control group. However, the control group had higher probability of weight loss from 0 to 1 kg. For females, the difference between maintaining weight and weight gain was not statistically significant. As for weight loss of more than 1 kg, the WeChat inactive group had lower probability than the control group. As for weight loss from 0 to 1 kg, the WeChat group (with both active and inactive subgroups) had lower probability than the control group (Table 5).

**Table 5:** Results of the multinomial logistic regression based on gender.

Gender and weight loss^a^				B	SE	Wald	*P*	OR (95% CI)
**Male**								
	**Weight unchanged**							
		Intercept		–0.08	0.44	0.03	.85	
		Propensity score		–1.68	0.67	6.20	.01	0.19 (0.05-0.70)
		**WeChat active^b^**						
			Activity (1)	1.10	0.26	17.47	<.001	3.01 (1.80-5.05)
			Inactivity (2)	1.34	0.18	58.70	<.001	3.84 (2.72-5.41)
	**Weight loss 0-1 kg**							
		Intercept		1.03	0.33	9.61	<.001	
		Propensity score		1.01	0.51	4.02	.045	2.75 (1.02-7.41)
		**WeChat active^b^**						
			Activity (1)	–0.62	0.19	10.61	<.001	0.54 (0.37-0.78)
			Inactivity (2)	–0.50	0.11	20.68	<.001	0.61 (0.49-0.75)
	**Weight loss 1-2 kg**							
		Intercept		1.22	0.36	11.62	<.001	
		Propensity score		–1.77	0.55	10.34	<.001	0.17 (0.06-0.50)
		**WeChat active^b^**						
			Activity (1)	0.75	0.21	13.09	<.001	2.12 (1.41-3.18)
			Inactivity (2)	0.90	0.13	49.17	<.001	2.46 (1.91-3.17)
	**Weight loss >2 kg**							
		Intercept		1.48	0.33	19.91	<.001	
		Propensity score		–0.73	0.51	2.06	.15	0.48 (0.18-1.30)
		**WeChat active^b^**						
			Activity (1)	0.61	0.19	10.93	<.001	1.85 (1.28--2.66)
			Inactivity (2)	0.53	0.11	21.69	<.001	1.69 (1.36--2.11)
**Female**								
	**Weight unchanged**							
		Intercept		–0.56	0.58	0.94	.33	
		Propensity score		0.61	0.68	0.79	.37	1.83 (0.48-6.97)
		**WeChat active^b^**						
			Activity (1)	0.10	0.21	0.24	.63	1.11 (0.73-1.68)
			Inactivity (2)	0.23	0.17	1.85	.17	1.26 (0.90-1.77)
	**Weight loss 0-1 kg**							
		Intercept		1.43	0.45	10.32	<.001	
		Propensity score		0.46	0.53	0.76	.38	1.59 (0.56-4.49)
		**WeChat active^b^**						
			Activity (1)	–0.41	0.16	6.26	.01	0.66 (0.48-0.91)
			Inactivity (2)	–0.62	0.13	21.93	<.001	0.54 (0.42-0.70)
	**Weight loss 1-2 kg**							
		Intercept		1.40	0.46	9.30	<.001	
		Propensity score		–0.31	0.55	0.32	.57	0.73 (0.25-2.14)
		**WeChat active^b^**						
			Activity (1)	–0.02	0.17	0.02	.89	0.98 (0.70-1.37)
			Inactivity (2)	–0.34	0.14	5.96	.02	0.71 (0.54-0.93)
	**Weight loss >2 kg**							
		Intercept		2.37	0.43	29.91	<.001	
		Propensity score		–0.86	0.52	2.78	.10	0.42 (0.15-1.16)
		**WeChat active^b^**						
			Activity (1)	–0.09	0.16	0.32	.57	0.91 (0.66-1.26)
			Inactivity (2)	–0.54	0.13	16.25	<.001	0.58 (0.45-0.76)

^a^ The reference category for the dependent variable was the classification as weight gain.

^b^ WeChat active was a subgroup variable, and the reference group was the control group.

## Discussion

This study proved that the weight loss intervention campaign, which was largely promoted by the Shunyi Government and based on an official WeChat account focused on an occupation-based population in Shunyi District, was very effective for males.

A total of 15,310 participants were enrolled in this study, among which 77.35% were willing to use WeChat for weight loss, which was consistent with a study that a WeChat health education program was evaluated with high levels of satisfaction from participants [23]. WeChat, one of the most popular mobile phone apps in China, may have significant potential to improve public health **.**

Participants in the WeChat group lost more weight (2.09 kg) on average than people in the control group (1.78 kg), and the difference in mean weight loss between the two groups for males was significant. For males, the results of the propensity score methods with a multinominal logistic regression showed that the WeChat group (with both active and inactive subgroups) had a higher probability of maintaining weight, weight loss from 1 to 2 kg, or weight loss more than 2 kg than the control group. However, the control group had higher probability of weight loss from 0 to 1 kg. Being active in WeChat is likely to be associated with weight loss. The more active participants were in the weight loss program via WeChat, the more weight they would lose.

Our WeChat intervention campaign provided participants with information on weight loss that could improve their knowledge, attitudes, practices, and so on. The results were in accordance with a previous study showing that participants’ knowledge, attitudes, skills, practices, and overall health literacy experienced greater changes via official WeChat accounts [23]. Our WeChat intervention campaign applied regular self-monitoring of physical activities, dietary intake, and weight, which played an important role in weight loss [28]. A study showed that specifically tailored text message reminders had no significant influence on weight loss among obese male employees for the possible reason that this intervention did not apply regular self-monitoring [29]. Moreover, our WeChat intervention campaign had interactive components such as the “microcommunity” component and the expert consultation component where people could get feedback and social support, which also played an important role in weight loss [30]. Social support was associated with weight loss in that the more positive social support, the greater the weight loss [31]. People have found positive social support for weight loss on Twitter [20]. Social support and information might be the two most common benefits of tweeting about weight loss. In addition, the WeChat intervention program might provide psychological benefits, where individuals can record their daily experiences, feelings, opinions, and so on [32], which might be useful for participants to lose weight.

The difference in mean weight loss between the two groups of females was not significant. WeChat might not affect or may even negatively affect weight loss for females. For females, the results of the propensity score methods with a multinominal logistic regression showed that the difference between maintaining weight and weight gain was not statistically significant. For the classification of weight loss more than 1 kg, the WeChat inactive group had lower probability than the control group. As for weight loss from 0 to 1 kg, the WeChat group (with both active and inactive subgroups) had lower probability than the control group. WeChat might have no effect on maintaining weight and might result in a lower chance of weight loss for females.

The WeChat intervention was effective on weight loss only for male employees. Females were more active using WeChat, but they lost less weight during the study. One reason might be that females were more motivated by the rewards than males and the rewards were given to the top 60 WeChat active participants per month, regardless of whether or not they in fact lost weight. Additionally, the fact that females spent more time on WeChat might negatively affect weight loss. One study showed that the more time a person spent on Facebook, the more negative feelings they had about their bodies due to more frequent body and weight comparisons for females [16]. Another explanation was that females might be more willing to lose weight but they did not prefer reporting personal information such as their weight and waist measurements publicly via WeChat. Thus, they were more active in WeChat but received less social support, feedback, and other benefits from WeChat. However, these people accounted for a large proportion of all participants. Thus, the difference between the two groups was not significant. In addition, males might have less offline social support and feedback than females, but males could obtain these benefits via our official WeChat account. Moreover, males were more competitive than females. In the future, interventions on weight loss, especially for females, should be developed with measures to protect personal privacy not with measures that collect private information in public. Additionally, a reward is a two-edged sword; in the future, WeChat activity as well as the amount of actual weight lost should be taken into consideration.

In the WeChat group, most of the participants were inactive in this study. It may be that participants showed greater interest in our WeChat intervention program at the beginning, but few people were able to adhere to our weight loss activities. The effectiveness of a weight loss intervention has been associated with intervention adherence [33]. Supervised attendance programs and interventions that offer social support result in higher adherence to weight loss activities [34]. Therefore, in the future, measures should be taken to improve adherence to our WeChat intervention program such as incorporating a supervisory component. Moreover, improving and refining the WeChat content is also very important. We must develop a variety of materials, not only articles but also more videos and cartoons on weight loss to attract interest. WeChat content should also be more authoritative and more concise. With the widespread use of WeChat and the large number of active users, WeChat may be a convenient, cost-effective medium to improve adherence to weight loss behaviors in China.

### Limitations

This study had some limitations. First, this study was a nonrandomized trial and although propensity score methods were used to control for confounding factors, the conclusions were limited. Second, weight loss behavior is a multifactorial phenomenon in that adherence [33], holidays, certain seasons, and important festivals [35], such as New Year’s Eve celebrations, may influence weight loss. Therefore, in the future, there should be greater focus on how to improve adherence to weight loss behaviors, and future studies over a longer period of time are necessary. Additionally, there was selection bias in the study.

### Conclusions

This weight loss intervention campaign based on an official WeChat account focused on an occupation-based population in Shunyi District was found to be effective for males. The more active male participants were in using WeChat, the more weight they lost. There might be no effects or even negative effects on weight loss for females. Future research should focus on how to improve adherence to the WeChat weight loss interventions and to protect personal privacy, especially for females.

## Abbreviations

BMI: body mass index
SYCDC: Shunyi Center for Disease Prevention and Control
